# Serum cytokine profiles in healthy nonhuman primates are blunted by sedation and demonstrate sexual dimorphism as detected by a validated multiplex immunoassay

**DOI:** 10.1038/s41598-021-81953-7

**Published:** 2021-01-27

**Authors:** Laura Hocum Stone, Scott Hunter Oppler, Julia L. Nugent, Sarah Gresch, Bernhard J. Hering, Michael P. Murtaugh, Rebecca L. Hegstad-Davies, Sabarinathan Ramachandran, Melanie L. Graham

**Affiliations:** 1 Department of Surgery, University of Minnesota, Minneapolis, MN 55455 USA; 2 Department of Veterinary Population Medicine, University of Minnesota, St. Paul, MN 55108 USA; 3 Veterinary Diagnostic Lab, College of Veterinary Medicine, University of Minnesota, St. Paul, MN 55108 USA; 4 Department of Veterinary and Biomedical Sciences, University of Minnesota, St. Paul, MN 55108 USA

**Keywords:** Preclinical research, Translational research

## Abstract

Cytokine profiling is a valuable tool for monitoring immune responses associated with disease and treatment. This study assessed the impact of sex and sedation on serum cytokines in healthy nonhuman primates (NHPs). Twenty-three cytokines were measured from serum using a bead-based multiplex assay. Assay validation for precision, sensitivity, recovery, linearity, and stability was performed. Samples from male and female cynomolgus and rhesus macaques either cooperating or sedated were compared. All cytokines except TNFα demonstrated acceptable sensitivity and precision, with variable recovery and linearity. IFNγ, IL-2, IL-5, IL-6, IL-8, IL-12/23 (p40), IL-13, IL-15, MCP-1, TGFα, VEGF met acceptance criteria; G-CSF, IL-4, IL-10, MIP1α, sCD40L were marginal. Higher cytokine levels were observed in females and cytokine levels were blunted in sedated NHPs when compared to awake cooperating NHPs. Significant differences observed in cytokines related to sex, species, or imposed by handling highlight the importance of model design on translational relevance for clinical settings.

## Introduction

Cytokines including chemokines, interferons, interleukins, growth factors, and tumor necrosis factors are mediators that play a critical regulatory role in inflammation and immunity. They exert their effects in shaping immunopathologic processes after secretion from primary cells by membrane-bound direct intercellular signaling. Cytokines are commonly used as important indicators of health, inflammation, and immune status. Since the discovery of the first interferon in 1957^1^ during studies of viral interference using influenza, the role of cytokines in the immune response to infectious diseases has been increasingly appreciated as a key contributor. The release of pro-inflammatory cytokines, classically IL-1, IL-6, and TNFα, to combat infection has a profound secondary effect on the overall physiologic system that can result in hypotension and tissue damage, as is seen in sepsis^2^. Additionally, recent studies have demonstrated a similar pro-inflammatory cytokine storm leading to ARDS after infection with SARS-CoV-2, the virus responsible for the global COVID-19 pandemic^3^. Interestingly, sedatives and anesthetics commonly used in critically ill and septic patients, as well as in surgical settings, have an anti-inflammatory effect thought to be due to a global reduction in pro-inflammatory cytokines and nitric oxide production^4,5^. This sedation effect may be beneficial in the treatment of sepsis, but also may drastically impact the interpretation of the immune response in cytokine-based studies where sedated or chemically restrained subjects are evaluated. This underappreciated impact of sedation and anesthetics on the immune system is important to consider when designing and evaluating scientific studies of changes in cytokine levels.

Cytokines transmit intracellular signals that are central to immune homeostasis or dysregulation and have a central role in pathologies like obesity and diabetes or cancer and autoimmunity. Conversely, the normal cytokine response can also be a target for immune manipulation like in the situation of transplantation where the host immune response to the graft must be suppressed to prevent rejection. In transplantation the achievement of immune tolerance reduces the risk of rejection and improves safety by eliminating toxic immunosuppression^6^. Immune profiling cytokines in health and disease has led to the development of numerous immunotherapy medications, both agonists and antagonists, for many diseases such as adult and juvenile rheumatoid arthritis, psoriasis, Inflammatory Bowel Disease, melanoma, colon cancer, lung cancer, renal cell carcinoma, multiple myeloma, and AIDS. For example, Adalimumab, an anti-TNFα antibody, has been used successfully to treat Inflammatory Bowel Disease^7^. IL-2 and IFNα in combination with chemotherapy have been used to treat metastatic melanoma, but development of many other cytokine treatments like IL-15 as single agents has not proceeded beyond Phase 2 trials^8^. Clinical inefficacy or negative side effects have so far limited the wide clinical success of cytokine-based therapies^9,10^, which is related to cytokine signaling across multiple pathways and many that exhibit redundancy and pleiotropy. The complexity of these targets drives the need for accurate and reliable measurements of cytokines to continue to advance the understanding of immune pathophysiology and immunomodulation strategies.

Commonly used animal models such as the mouse have high value as screening or mechanistic models due to the wealth of information that can be obtained from genetic modifications and the availability of reagents. Nonhuman primates (NHPs) are used to bridge gaps that cannot be addressed by other animal models and because of high predictive validity are among the most robust translational model for infectious disease, neuroscience, transplantation, and reproductive biology in humans. The outbred nature of NHPs as well as anatomy^11,12^ and educated immune system similar to that of humans makes the NHP a uniquely relevant model system that allows for valid modeling of human diseases, as well as for safety and efficacy studies of drugs, and implantable devices^13,14^. Humans and old-world primates, a group which includes cynomolgus and rhesus macaques, diverged from a common ancestor approximately 25 million years ago^15^ and macaques share 93% of their genome with humans^16^. One such example includes the genetic similarity between NHPs and humans in immunologically important MHC class I and II regions^15^. Additionally, the expression of Toll-Like Receptors within subsets of dendritic cells are analogous in humans and NHPs but not rodents, which has far-reaching implications for innate immune function^13^. In immune senescence research, a hallmark loss of CD28 expression by T cells in humans is accurately modeled by NHPs but not by rodents^13^. When using NHPs in preclinical testing, accurate, reproducible, and independently validated measurements of longitudinal and clinically relevant endpoints, such as levels of cytokines in serum, are critical. Unfortunately, validation reports supporting the reliability of methods for the measurement of cytokines in NHP serum are rare, and those that have been published do not have uniform acceptance criteria and validation methods^17–19^, making interpretation between studies challenging.

Multiplexed cytokine assays have gained great popularity in both human and animal research due to their ability to provide a more complete picture of the immune response, which is important given the complex interactions of the cytokine network in both disease progression as well as regulation of disease. Most assays in current use are similar to a traditional ELISA, but with each capture antibody coated onto individual beads which are then combined for the assay, allowing for the measurements of double-digit numbers of cytokines. Multiplex assays have the comparative advantage of small sample volume relative to the circulating blood volume in the NHP, making frequent serial sampling possible without compromising welfare to investigate disease progression from the subtleties to potential major biomarkers. While many studies report cytokine measurements from NHP blood using multiplex assays, there is a lack of method validation for multiplex cytokine assays in NHP model in the current literature. Method validation is necessary to account for any inherent methodological error and study limitations, and is critical to appropriately interpret reported values from the assay. Method validation procedures include the demonstration of analytical sensitivity, linearity, precision (intra- and inter-assay), and accuracy (the ability to recover a spiked analyte). Multiplex assays pose a unique challenge for validation given the myriad of possibilities for interference and cross-reactivity of so many analytes as well as the technical challenges of a complex bead-based assay. In 2014, He et al. described the only known validation of a multiplex cytokine assay in NHPs^20^. Unfortunately, with the rapid improvements in cytokine multiplexing technology, the assays in this study are no longer available. Additionally, this study did not report intra- or inter-assay variability, which can play a large role in the quantitative results obtained.

We sought to independently validate a commonly-used, commercially-available multiplex cytokine assay for NHPs by establishing acceptance criteria for cytokine measurements by determining which cytokines in the assay met said criteria to ensure experimental rigor and support the proper evaluation of nonclinical laboratory studies subject to FDA, EMA, and ICH guidance. Once validated, cytokine concentrations were measured in serum from male and female sedated and cooperating cynomolgus and rhesus macaques. To determine if these sex and model handling variables are associated with baseline secretory patterns, we collected laboratory data retrospectively after the introduction of the validated assay. While such observations have been demonstrated in other species, they have not previously been made in macaques, a highly relevant animal model for preclinical testing. This is of interest because often a change from baseline levels or the balance between pro- and anti-inflammatory cytokines is what is associated with disease progression or improvement^21^. We evaluated cytokine secretion patterns in two NHP species (*Macaca fascicularis,* cynomolgus macaques*,* and *Macaca mulatta,* rhesus macaques) commonly used as models of infectious disease, neurodegenerative disorders, and regenerative medicine. Understanding and being able to reliably measure cytokine levels in healthy NHPs as a translational animal model is critical for future research into cytokine effects and roles in disease, allowing for the development of more personalized, robust, and effective therapeutics.

## Methods

### Animal subjects

All animal procedures were approved by the University of Minnesota Institutional Animal Care and Use Committee, conducted in compliance with the Animal Welfare Act, adhered to principles stated in the Guide for Care and Use of Laboratory Animals published by the US National Institutes of Health (NIH publication no 85-23), and studies performed and reported in compliance with the ARRIVE guidelines.

A total of 93 healthy cynomolgus macaques (*Macaca fascicularis*) (female = 29, male = 64) and 26 healthy rhesus macaques (*Macaca mulatta*) (female = 5, male = 21) were evaluated in this study (Supplemental Table 1 shows cohort demographics). All animals were purpose-bred and purchased from institutionally approved commercial vendors. They were housed in pairs or small groups of the same sex. They had free access to water and were fed biscuits (Purina No. 5408, St Louis, MO, USA, or Harlan Teklad Global Diets‐Primate 2055, Madison, WI, USA) based on body weight, supplemented with fresh fruits, vegetables, grains, beans, nuts, and a multivitamin preparation. The animals participated in an environmental enrichment program that included social play, opportunities for foraging, puzzle solving, music, and regularly scheduled access to exercise and swimming areas. Semi-annual veterinary physical examinations were performed and included weight, body condition scoring, heart rate, temperature, palpation of lymph nodes and abdomen, and evaluation of the oral cavity, dermis, ears and nose, as well as a complete blood count and chemistry panel. Animals were concurrently enrolled in separate metabolic studies with exclusion criteria that included body weight under 1.8 kg, severe persistent diarrhea or vomiting, active systemic infection, lymphopenia, neutropenia, thrombocytopenia, elevated hemoglobin or liver enzymes or plasmodium infection. To facilitate cooperative blood sampling, drug administration, fluid infusion, and simple physical examination, animals were trained using a positive reinforcement paradigm to cooperatively present their VAP in the home cage which removed the need for physical or chemical restraint for these routine procedures^22,23^. NHPs were observed at least twice daily for general appearance, temperament, social interaction, gait, urination, defecation, and body condition as a part of routine health monitoring.

### Sample collection

At time of sampling, cynomolgus macaques were aged between 3.0 and 12.9 years (median 5.1 years, IQR 4.5–5.8 years), and weighed between 3.11 and 7.94 kg (median 4.76 kg, IQR 4.1–5.7 kg). Rhesus macaques were aged between 1.7 and 13.1 years (median 3.9 years, IQR 3.5–4.4 years), and weighed between 3.05 and 16.2 kg (median 6.14 kg, IQR 3.9–7.3 kg). Blood samples were collected from awake, cooperating animals (n = 90; n = 64 cynomolgus macaques; n = 26 rhesus macaques) via indwelling vascular access port^24^ or standard venipuncture from the cephalic, saphenous, or femoral vein, and from sedated animals (n = 29, cynomolgus macaques only). Animals were sedated with either 8–10 mg/kg ketamine (IM) (n = 27) or 4 mg/kg tiletamine combined with zolazepam (IM) (n = 2). The tiletamine/zolazepam-sedated animals were excluded from analyses comparing cytokine expression in cooperating and sedated NHPs. Of the 27 animals, 5 were sedated with ketamine only, and the remaining 22 were sedated with ketamine only (n = 11) or ketamine in combination with 0.1–0.3 mg/kg midazolam (n = 11) for anesthesia induction with inhalation isoflurane (Supplemental Table 1). Blood used for cytokine evaluation (0.6 mL) was collected prior to any procedural manipulation in Becton Dickinson serum separator microtainer tubes. Tubes were kept cool and allowed to clot for 30 min then centrifuged at 3287×*g* for 20 min. Samples were aliquoted into plastic microfuge tubes and frozen at − 80 °C until use. Additional blood was collected in EDTA tubes (0.5 mL) for CBC evaluation, and serum separator tubes (0.6 mL) for chemistry evaluation.

### Milliplex cytokine measurement

Millipore Milliplex MAP premixed bead-based 23-plex kits for Non-Human Primate Cytokines (#MPXPRCYTO-40K-PX23, #PCYTMG-40K-PX23) were used according to manufacturer instructions^25^. Change in kit number was due to manufacturer transition in kit format from polystyrene to magnetic beads. All validation-related measures were performed using the magnetic bead-based kit. These kits includes premixed beads for 23 cytokines: G-CSG, GM-CSF, IFNγ, IL-1β, IL-1ra, IL-2, IL-4, IL-5, IL-6, IL-8, IL-10, IL-12/23(p40), IL-13, IL-15, IL-17A, MCP-1, MIP-1α, MIP-1β, sCD40L, TGFα, TNFα, VEGF and IL-18. Standard curves for each cytokine were prepared by serial dilution and run in triplicate (Polystyrene Bead Kit: 0.64–10,000 pg/mL for all cytokines; Magnetic Bead Kit: IL-4: 4.9–20,000 pg/mL; IL-10 and IL-18: 12.2–50,000 pg/mL; remaining cytokines: 2.4–10,000 pg/mL). The standards and controls for nine of the cytokines (IFNγ, IL-1β, IL-2, IL-4, IL-5, IL-12, IL-13, TNFα and IL-18) were recombinant NHP proteins; the standards and controls for the other 14 cytokines were human proteins. The detecting antibodies for IL-2 were generated against monkey (unspecified species) protein; the detecting antibodies for VEGF were generated against mouse protein; the detecting antibodies for the remaining 21 cytokines were generated against human proteins. The manufacturer reports “no or negligible” cross-reactivity between any of the analytes of the assay panel. A human serum matrix provided with each assay kit is added to the standard and control wells to mimic the NHP matrix of the samples. A Luminex100 system equipped with xPONENT v. 3.1 software was used to perform the multiplexed assays.

### Validation assays

#### Study design

Samples from 20 cynomolgus macaques were pre-selected as naïve and required sampling for other enrolled studies, allowing for opportunistic sampling for this study. These 20 animals were used for validation testing of the multiplex assay. All validation measurements were performed across four assay plates.

#### Recovery

To evaluate recovery (measured as observed concentration relative to expected concentration), eight unique serum pool samples were made from multiple samples taken from a single animal on different days, with the exception of one of the serum pools was constructed from samples of two different animals due to sample availability. Each sample was measured in three ways: neat (unspiked) and with spiking of a known amount of either of two manufacturer provided standards (highest concentration—kit “standard 7”—and mid-range concentration—kit “standard 5”). Each spiked sample consisted of 75% endogenous NHP serum and 25% kit standard (Supplemental Table 2). Each sample was tested in triplicate.

#### Linearity

Linearity (measured as observed concentration relative to the expected concentration in a sample with known dilution) was evaluated using the same neat serum pool samples used in for the evaluation of assay recovery. Each sample was spiked with the highest concentration kit standard and then measured at 1:2 and 1:5 assay buffer dilutions. Three of the eight samples were also measured neat (unspiked) and at a 1:2 dilution (Supplemental Table 2). Each sample was tested in triplicate.

#### Precision

To evaluate intra-assay precision, two unique serum pools were made from multiple samples taken from a single animal on different days. Pooled serum was spiked with a known amount of either a mid-range (kit “standard 5”) or high-concentration (kit “standard 7”) kit-provided standard to determine precision at limits. Each spiked sample was constructed such that > 90% of the final spiked matrix consisted of endogenous NHP serum. Each sample was tested with 12 replicates within a single assay plate (Supplemental Table 3A). To evaluate inter-assay precision, four control samples were each measured in triplicate across four assay plates. Two controls were manufacturer-provided (lot-controlled), and two controls were made from NHP serum pools spiked with a known amount of either of the two manufacturer-provided controls at a 1:4 assay buffer dilution. These NHP control pools were aliquoted into single-use tubes and frozen until use to avoid additional freeze/thaw cycles. Each spiked sample consisted of 50% endogenous NHP serum and 50% manufacturer-provided control (Supplemental Table 3B).

#### Sensitivity

To evaluate assay sensitivity, the lowest kit-provided standard (kit “standard 1”) was measured at 1:2 and 1:4 assay buffer dilutions to extend each analyte’s standard curve. Both the lower limit of detection (LLOD, the lowest concentration that can be detected) and lower limit of quantification (LLOQ, the lowest concentration that can be precisely and reliably detected) were determined for each cytokine, though only the LLOD was used to determine if validation acceptance criteria were met (LLOD ≤ the lowest standard). Each dilution was tested on a single assay plate six times (Supplemental Table 4).

#### Within-subject variability

Variability was evaluated by measuring cytokine values in repeated sampling from the same subject on different days. The ratio of the highest level measured to the lowest level measured for each cytokine was calculated to assess variability across these samples.

### In vitro ketamine effects

To evaluate the effects of ketamine on assay performance (versus in vivo physiologic effects), three serum samples, each taken from a different cooperating cynomolgus macaque were measured neat (unspiked), and spiked with ketamine. Ketamine was diluted to 0.02 mg/mL with kit-provided sample matrix, and added to serum samples to give a final concentration of 2000 ng/mL, the reported peak concentration of ketamine in human plasma during sedation^26^. Samples were tested in duplicate on a single assay plate.

### Data analysis

Cytokine concentration was determined by fluorescence intensity. Fluorescence data were analyzed with Millipore Milliplex Analyst version 3.4 according to manufacturer recommendations. All statistical analyses were performed with the calculated concentrations of each sample rather than the MFI (mean fluorescence intensity) source data to give a more accurate representation of assay performance.

Assessment of intra- and inter-assay precision was measured by the coefficient of variation (CV%) calculated from the measured concentration of each replicate. Measurements of sensitivity, recovery, and linearity were calculated as a ratio of the assay returned cytokine value compared to the expected cytokine value.

Statistical analysis was performed using GraphPad Prism version 8.4.1 (San Diego, CA). Outliers were identified using the Tukey method but were not excluded. Data points falling below the LLOD were adjusted to half the value of the LLOD for that cytokine (i.e. < 0.64 was changed to 0.32)^27^. Significance between two NHP groups was determined using non-parametric unpaired, two-way Mann–Whitney tests with α = 0.05. Significance between the neat and ketamine-spiked serums samples was determined using non-parametric Wilcoxon matched pairs signed rank tests with α = 0.05. Multivariate analysis was conducted using a two-way ANOVA with Box-Cox transformed data to meet statistical assumptions. The two-way ANOVA table was generated using software from Assaad et al.^28^. All data sets were tested for normality using the Kolmogorov–Smirnov test, and unequal variance was assessed using the F-test.

## Results

### Validation

#### Recovery

**Figure 1:**
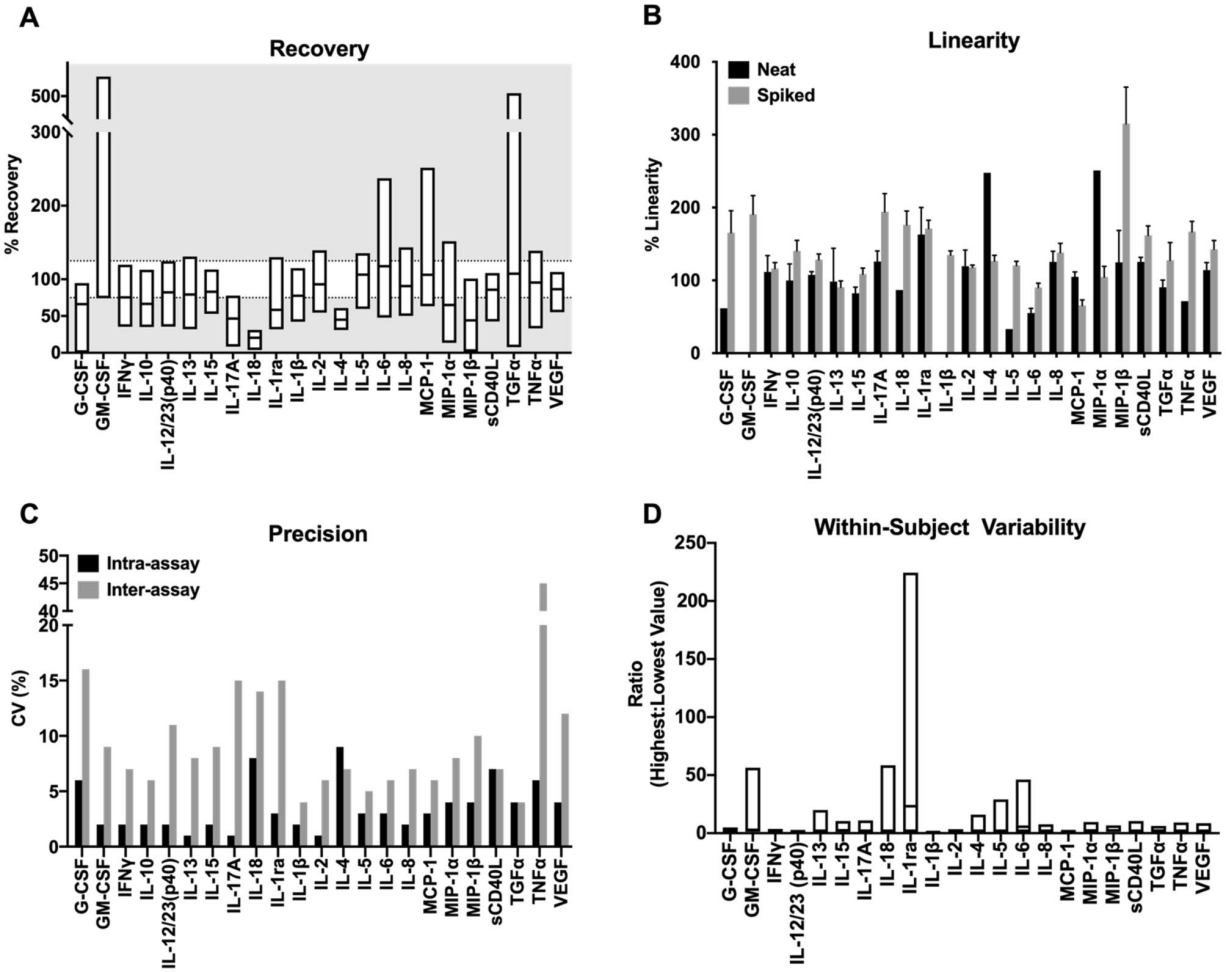
Validation study results. (**A**) Analyte recovery. NHP serum was spiked with a known amount of each cytokine. Recovery values between 75 and 125% were considered acceptable. (**B**) Linearity. NHP serum was tested neat as well as spiked with a known concentration of each cytokine. Linearity ranges between 75 and 125% were considered acceptable. (**C**) Precision. Intra-assay precision was measured between duplicate samples (n = 24). Inter-assay precision was measured across technical replicates (n = 4). (**D**) Within-Subject Variability. Variability was measured by calculating the variability in cytokine measurements between samples taken from the same animal.

Final recovery acceptance criteria for a given cytokine was defined as more than half of all samples for a given analyte meeting the acceptance criteria of 75–125% of the expected value. Thirteen of 23 cytokines met validation acceptance criteria for recovery (Fig. 1A, Supplemental Table 5).

For several analytes (TGFα, IFNγ, IL-10, MIP1α, and TNFα), recovery performance varied considerably across the samples. For example, in one assay, TGFα showed an average recovery of 114% in one animal, but an average recovery of 70% in another. In contrast, three analytes (IL-5, IL-8 and MCP-1) were consistently recovered across animals and assays—each had acceptable recovery levels across all determinations. GM-CSF consistently had a recovery of between 200 and 300%. Four analytes (IL-17A, IL-4, MIP-1β and IL-18) showed consistently low recoveries across animals and assays (Supplemental Table 5).

#### Linearity

Final linearity acceptance criteria for a given cytokine was defined as more than half of all samples, or all diluted neat samples for a given analyte falling in the acceptable 75–125% recovery range. Fourteen of 23 cytokines met validation acceptance criteria for linearity (> 50% of the samples demonstrated acceptable average linearity AND/OR overall acceptable linearity (75–125%) of endogenous (neat) samples) (Fig. 1B, Supplemental Table 6). All three samples that were tested neat had undetectable levels of GM-CSF and IL-1β, therefore no linearity data could be gathered for those analytes until the samples were spiked.

### Precision

The pre-established acceptance criteria for a multiplexed method is CV < 25% for inter-assay precision and < 20% for intra-assay precision (based on common published parameters^18,19,29^). All cytokines met acceptance criteria of CV < 20% for intra-assay precision (Fig. 1C, Supplemental Table 7). Inter-assay CV was acceptable (< 25%) for 22 of 23 cytokines; TNFα showed an unacceptable inter-assay CV of 45.3% (Fig. 1C, Supplemental Table 7). As expected, inter-assay CVs were generally higher than intra-assay CVs, demonstrating the day-to-day variability of a technically challenging assay such as this one. In comparison to the manufacturer-reported intra-assay precision, the current study agreed with the manufacturer reported CV of < 5% in 18 of 23 (78%) analytes for intra-assay precision and the manufacturer-reported CV of < 15% in 20 of 23 (87%) analytes for inter-assay precision (Fig. 1C).

### Sensitivity

In order to meet assay acceptance criteria, it is expected that the LLOD will be at, or below, the lowest standard for that analyte. The results of the sensitivity studies (Supplemental Table 8) show that this assay is sensitive at or below the lowest standard for all 23 cytokines method.

### Within-subject variability

To determine the stability of detectable cytokine values across assays, within-subject variability was calculated by the ratio of the highest level measured to the lower level measured of each cytokine taken from multiple samples in the same animal on three different days. IL-1ra had the highest variability, with a mean intra-animal ratio of 23.23. The remaining cytokines had an average stability ratio of 3.08 (Fig. 1D).

### Acceptance criteria

Each of the 23 cytokines were categorized as acceptable, marginal, or rejected based upon validation acceptance criteria for sensitivity, precision, recovery, and linearity^30,31^. All of the cytokines met the acceptance criteria for sensitivity and intra-assay precision, and all but one cytokine (TNFα) met the acceptance criteria for inter-assay precision. Validation acceptance criteria was more varied for recovery and linearity. Final recovery and linearity acceptance classification for each cytokine are summarized in Table 1. If conditions were met for all validation acceptance criteria, the cytokine was categorized as having acceptable validity using the multiplex method (11 out of 23; 48%). If conditions were met for all but either recovery or linearity, the cytokine validity was categorized as marginal (5 out of 23; 22%). If more than one condition was not met, the cytokine validity was classified as rejected (7 out of 23; 30%).

**Table 1:** Validation outcomes for cytokines measured in healthy NHP serum.

Cytokine	Precision	Sensitivity	Recovery	Linearity	Validation outcome
*G-CSF*	*Pass*	*pass*	*Pass*	*Fail*	*Marginal*
**GM-CSF**	**Pass**	**pass**	**Fail**	**Fail**	**Reject**
IFNγ	Pass	Pass	PASS	Pass	Accept
*IL-10*	*Pass*	*Pass*	*Fail*	*Pass*	*Marginal*
IL-12/23 (p40)	Pass	Pass	Pass	pass	Accept
IL-13	Pass	Pass	Pass	Pass	Accept
IL-15	Pass	Pass	Pass	Pass	Accept
**IL-17A**	**Pass**	**Pass**	**Fail**	**Fail**	**Reject**
**IL-18**	**Pass**	**Pass**	**Fail**	**Fail**	**Reject**
**IL-1ra**	**Pass**	**Pass**	**Fail**	**Fail**	**Reject**
**IL-1β**	**Pass**	**Pass**	**Fail**	**Fail**	**Reject**
IL-2	Pass	Pass	Pass	Pass	Accept
*IL-4*	*Pass*	*Pass*	*Fail*	*Pass*	*Marginal*
IL-5	Pass	Pass	Pass	Pass	Accept
IL-6	Pass	Pass	Pass	Pass	Accept
IL-8	Pass	Pass	Pass	Pass	Accept
MCP-1	Pass	Pass	Pass	Pass	Accept
*MIP-1α*	*Pass*	*Pass*	*Fail*	*Pass*	*Marginal*
**MIP-1β**	**Pass**	**Pass**	**Fail**	**Fail**	**Reject**
*sCD40L*	*Pass*	*Pass*	*Pass*	*Fail*	*Marginal*
TGFα	Pass	Pass	Pass	Pass	Accept
**TNFα**	**Pass**	**Pass**	**Fail**	**Fail**	**Reject**
VEGF	Pass	Pass	Fail	Pass	Accept

Regular text = accept; italicized = marginal; bold = reject.

### Female cynomolgus, but not rhesus, macaques have a more dynamic range of serum cytokine expression than males

**Figure 2:**
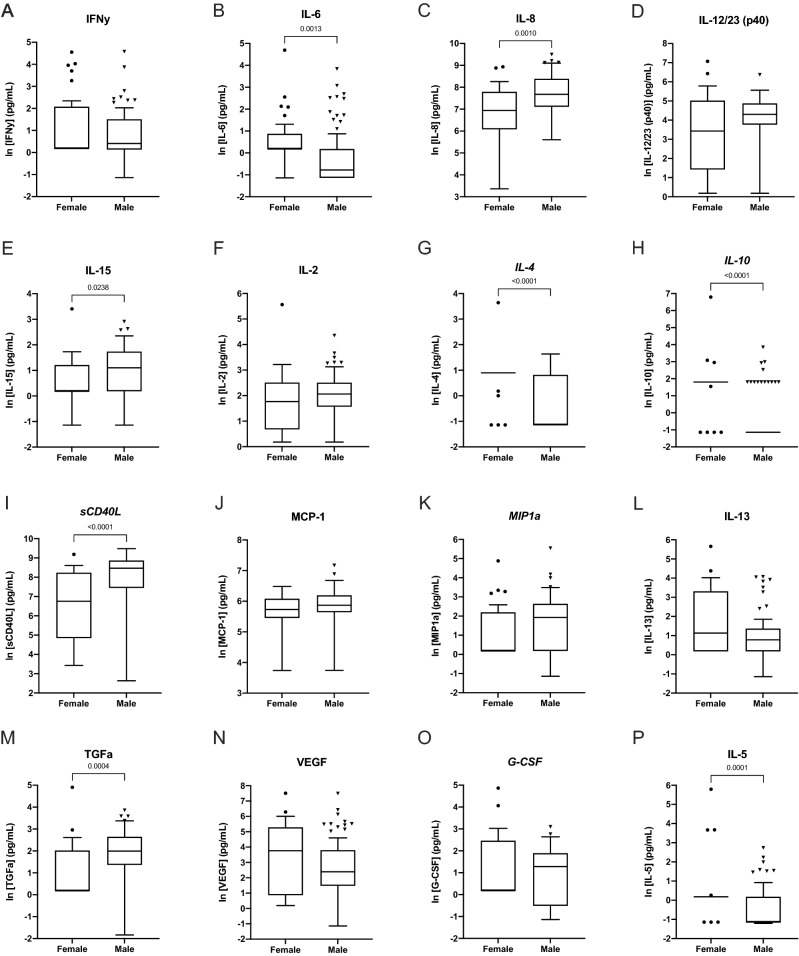
Cytokine detection by sex in cynomolgus macaques. Box-plots of serum cytokine detection (pg/mL) in female and male cynomolgus macaques. Whiskers on box-plots were calculated using the Tukey method. P-values representing significant group differences were calculated based on non-parametric, unpaired two-way Mann–Whitney tests. Regular title = accept; italicized title = marginal.

Detectable cytokine ranges were determined for male and female cohorts of both cynomolgus and rhesus macaques (Table 2); these cohorts included both cooperating and sedated animals pooled together to first evaluate differences by species and sex. The number of samples which fell within the dynamic range of the assay in healthy animals varied greatly by cytokine, with some having more than half of samples falling below the LLOD. The detection ranges for each cytokine varied both by species and by sex. In cynomolgus macaques, cytokines varied significantly by sex in both pro- and anti-inflammatory roles, with females exhibiting a greater dynamic range of cytokine expression among the majority of cytokines in comparison with males (Table 2, Fig. 2). The cytokine levels detected between cynomolgus macaques and rhesus macaques (Table 2) were quite different, demonstrating the importance of species-specific analysis. The sex-based differences seen in cynomolgus macaques did not hold true in the rhesus macaques (Table 2, Fig. 2), although the sample size is smaller, possibly influencing these results. Within same-sex cohorts, cytokine levels did not differ significantly between animals less than and greater than 5 years of age, with the exception of IL-12/23 expression in cynomolgus macaque males only (p = 0.006). Males less than 5 had a median IL-12/23 expression of 66.5 (IQR 8.2–96.8) pg/mL, while males greater than 5 had a median IL-12/23 expression of 85.9 (60.5–161.2) pg/mL.

**Table 2:** Cytokine detection in all NHPs, cynomolgus, and rhesus macaques.

**Cytokine**	All		Cynomolgus		Rhesus	
	N	Median (IQR) (pg/mL)	N	Median (IQR) (pg/mL)	N	Median (IQR) (pg/mL)
**Major pro-inflammatory roles**						
IFNy						
All	119	1.2 (1.2–3.8)	93	1.2 (1.2–4.9)	26	1.2 (1.2)
Female	34	1.2 (1.2–6.7)	29	1.2 (1.2–8.0)	5	1.2 (1.2)
Male	85	1.2 (1.2–3.8)	64	1.5 (1.1–4.5)	21	1.2 (1.2)
P value		0.5290		0.615		> 0.9999
IL-6						
All	119	1.2 (0.3–1.2)	93	1.2 (0.3–1.2)	26	1.2 (1.2)
Female	34	1.2 (1.2–1.3)	29	1.2 (1.2–2.4)	5	1.2 (1.2)
Male	85	1.2 (0.3–1.2)	64	0.5 (0.3–1.2)	21	1.2 (1.2)
P value		0.0071**		0.0013**		0.5962
IL-8						
All	119	1736.0 (804.9–4102.0)	93	1707.0 (795.5–3897.0)	26	1867.0 (846.2–4317.0)
Female	34	980.7 (378.2–2538.0)	29	1032.0 (436.6–2423.0)	5	266.5 (67.9–5794.0)
Male	85	2147.0 (1010.0–4353.0)	64	2163.0 (1223.0–4396.0)	21	1932.0 (930.8–4362.0)
P value		0.0005***		0.001**		0.3083
IL-12/23 (p40)						
All	119	50.9 (4.8–107.8)	93	69.8 (15.5–133.8)	26	4.4 (1.2–29.2)
Female	34	14.2 (3.4–95.1)	29	31.0 (4.2–151.1)	5	1.2 (1.2–13.4)
Male	85	62.7 (17.9–110.3)	64	73.8 (43.1–130.4)	21	5.0 (1.2–36.1)
P value		0.1334		0.0918		0.2343
IL-15						
All	119	3.1 (1.2–5.9)	93	2.6 (1.2–5.1)	26	6.4 (3.3–7.2)
Female	34	1.2 (1.2–3.5)	29	1.2 (1.2–3.4)	5	3.4 (2.0–10.1)
Male	85	3.6 (1.5–6.6)	64	3.0 (1.2–5.7)	21	6.6 (4.0–7.3)
P value		0.0037**		0.0238*		0.2500
**Major anti-inflammatory roles**						
IL-2						
All	119	7.3 (3.7–12.5)	93	7.3 (4.1–12.3)	26	7.4 (2.8–18.0)
Female	34	6.2 (2.4–12.8)	29	5.9 (2.0–12.3)	5	17.0 (2.9–18.7)
Male	85	7.6 (4.4–12.9)	64	7.9 (4.8–12.3)	21	6.3 (2.7–18.1)
P Value		0.3150		0.1514		0.7042
*IL-4*						
* All*	*114*	*2.5 (0.3–2.5)*	*88*	*0.3 (0.3–2.5)*	*26*	*2.5 (2.5)*
* Female*	*31*	*2.5 (2.5)*	*26*	*2.5 (2.5)*	*5*	*2.5 (2.5–1408.0)*
* Male*	*83*	*0.3 (0.3–2.5)*	*62*	*0.3 (0.3–2.3)*	*21*	*2.5 (2.5)*
* P value*		0.0002***		< 0.0001****		*0.6664*
*IL-10*						
* All*	*119*	*6.1 (0.3–6.1)*	*93*	*0.3 (0.3–6.1)*	*26*	*6.1 (6.1)*
* Female*	*34*	*6.1 (6.1)*	*29*	*6.1 (6.1)*	*5*	*6.1 (6.1)*
* Male*	*85*	*0.3 (0.3–6.1)*	*64*	*0.3 (0.3)*	*21*	*6.1 (6.1)*
* P value*		< 0.0001****		< 0.0001****		*0.5552*
*sCD40L*						
* All*	*119*	*3491.0 (483.1–5576.0)*	*93*	*3851.0 (705.9–5867.0)*	*26*	*971.3 (331.9–5007.0)*
* Female*	*34*	*952.0 (164.3–4238.0)*	*29*	*860.5 (127.4–3786.0)*	*5*	*5860.0 (2877.0–9308.0)*
* Male*	*85*	*4093.0 (553.5–6476.0)*	*64*	*4768.0 (1712.0–7116.0)*	*21*	*483.1 (319.0–4338.0)*
* P value*		0.0071**		< 0.001****		0.0160*
**Macrophage/T-cell recruitment roles**						
MCP-1						
All	119	328.7 (229.8–459.2)	93	338.2 (271.9–463.8)	26	237.9 (178.9–349.1)
Female	34	289.7 (212.2–435.4)	29	307.9 (233.6–438.9)	5	219.3 (191.2–402.1)
Male	85	336.3 (240.8–480.9)	64	353.2 (281.5–490.1)	21	246.1 (178.2–364.3)
P value		0.1364		0.0578		0.9498
*MIP-1a*						
* All*	*119*	*4.3 (1.2–11.3)*	*93*	*5.9 (1.2–11.6)*	*26*	*1.2 (1.2–3.8)*
* Female*	*34*	*1.2 (1.2–8.5)*	*29*	*1.2 (1.2–9.0)*	*5*	*1.2 (1.2–4.7)*
* Male*	*85*	*5.4 (1.2–12.2)*	*64*	*6.9 (1.2–14.0)*	*21*	*1.2 (1.2–4.4)*
* P value*		*0.2100*		*0.1446*		*0.7348*
IL-13						
All	119	2.4 (1.2–6.5)	93	2.2 (1.2–6.3)	26	3.9 (1.2–8.1)
Female	34	3.1 (1.2–25.5)	29	3.1 (1.2–27.4)	5	3.7 (1.2–7.0)
Male	85	2.3 (1.2–5.5)	64	2.2 (1.2–3.9)	21	4.2 (1.2–13.0)
P value		0.3639		0.2455		0.5284
**Wound healing/miscellaneous**						
TGFα						
All	118	4.4 (1.2–10.5)	92	5.6 (1.5–12.0)	26	1.8 (1.2–4.9)
Female	33	1.2 (1.2–5.7)	28	1.2 (1.2–7.6)	5	2.9 (1.2–3.7)
Male	85	5.4 (2.6–11.8)	64	7.3 (3.9–14.1)	21	1.2 (1.2–5.1)
P value		0.0017**		0.0004***		0.7905
VEGF						
All	117	16.5 (4.3–81.8)	93	12.2 (4.3–92.5)	24	46.5 (5.8–77.8)
Female	34	38.1 (2.2–158.9)	29	42.7 (2.4–196.2)	5	33.5 (1.9–65.2)
Male	83	15.8 (4.5–70.2)	64	10.9 (4.4–44.7)	19	62.1 (15.8–142.6)
P value		0.2010		0.0824		0.4443
*G-CSF*						
* All*	*96*	*2.3 (1.2–7.1)*	*79*	*3.1 (1.2–7.2)*	*17*	*1.2 (1.2–4.6)*
* Female*	*29*	*1.2 (1.2–11.7)*	*24*	*1.2 (1.2–11.7)*	*5*	*1.2 (1.2–10.4)*
* Male*	*67*	*2.9 (1.2–6.0)*	*55*	*3.6 (0.6–6.6)*	*12*	*1.2 (1.2)*
* P value*		*0.3068*		*0.4595*		*0.4916*
IL-5						
All	119	1.2 (0.3–1.2)	93	1.2 (0.3–1.2)	26	1.2 (1.2)
Female	34	1.2 (1.2)	29	1.2 (1.2)	5	1.2 (1.2)
Male	85	1.2 (0.3–1.2)	64	0.3 (0.3–1.2)	21	1.2 (1.2)
P value		0.0020**		0.0001***		> 0.9999

Regular text = accept; italicized = marginal. Cytokines with validation outcome of reject are not included.

*p < 0.05; **p < 0.01; ***p < 0.001; ****p < 0.0001.

### Sedation results in an overall blunting of serum cytokine expression in cynomolgus macaques

**Figure 3:**
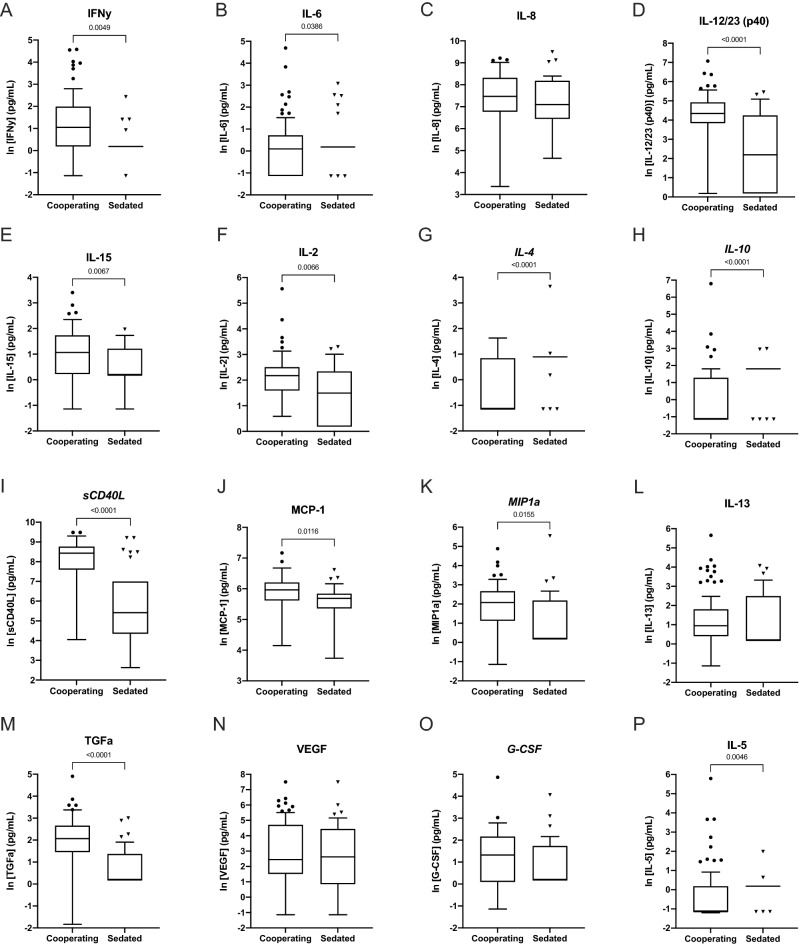
Cytokine detection in cooperating and sedated cynomolgus macaques. Box-plots of serum cytokine detection (pg/mL) in cooperating and sedated cynomolgus macaques. Whiskers on box-plots were calculated using the Tukey method. P-values representing significant group differences were calculated based on non-parametric, unpaired two-way Mann–Whitney tests. Regular title = accept; italicized title = marginal.

To assess the effect of sedation on cytokine detection, detectable ranges were also determined for sampling-method subgroups (cooperating vs. sedated) cynomolgus macaques (Fig. 3, Supplemental Table 9); rhesus macaques were not evaluated due to small sample numbers. Nearly all measured cytokines showed a significant reduction in detectable concentration in sedated animals as compared to awake, with the exception of IL-8 (similar levels), MIP-1α (slightly increased after sedation), IL-13 (similar levels), VEGF (slightly increased after sedation), G-CSF (similar levels). This suggests an overall blunting effect of sedation on cytokine expression in cynomolgus macaques. Of note, we spiked ketamine directly into three samples to assess if this suppressed cytokine profile was due to a direct interaction of ketamine within the assay. This showed no significant difference in neat (unspiked) compared to spiked samples (Supplemental Table 10), indicating that sedation does blunt the physiologic systemic cytokine response.

To further investigate the interaction between sex and sedation on cytokine expression, we compared the cytokine expression of each cohort subset—cooperating females, sedated females, cooperating males, and sedated males (Table 3). We found that the blunted cytokine response seen with sedation persisted in both males and females, but this effect was more dramatic in female subjects, perhaps due to their more dynamic ranges of detection. Differences in cytokine expression between sexes was most apparent when comparing cooperating animals, as sedated animals of both sexes demonstrated a suppressed cytokine response. Overall, subject handling had a significant effect on a higher number of cytokines than sex. These two variables had a significant interaction for the expression of IL-5, IL-8, IL-10, and G-CSF.

**Table 3:** Multivariate analysis of the impact of sex and handling variables on cytokine expression demonstrated that among cooperating cynomolgus macaques, males and females demonstrated a significantly different cytokine expression profile.

Variable	Female		Male		*P*-value		
	Cooperating (n = 16)	Sedated (n = 13)	Cooperating (n = 48)	Sedated (n = 14)	Sex	Handling	S1 × S2^1^
**Major pro-inflammatory roles**							
IFNγ	194 ± 78.8^b^	42.2 ± 17.7^a^	104 ± 12.7^ab^	45.5 ± 19.3^ab^	0.294	0.012*	0.072
IL-6	8.61 ± 6.74^b^	2.96 ± 1.02^b^	2.88 ± 1.04^a^	3.27 ± 1.63^ab^	0.001**	0.151	0.306
IL-8	2180 ± 582^a^	1060 ± 277^b^	2920 ± 368^ab^	3990 ± 1020^ab^	0.661	0.148	0.018*
IL-12/23 (p40)	38.5 ± 17.5^a^	7.88 ± 3.41^b^	5.71 ± 1.64^ab^	10.4 ± 5.11^b^	0.234	< 0.001**	0.259
IL-15	19.6 ± 6.9	1.2 ± 0	6.43 ± 2.21	2.38 ± 0.756	0.194	0.063	0.64
**Major anti-inflammatory roles**							
IL-2	24 ± 15.8^a^	6.75 ± 2.39^b^	11.4 ± 1.78^a^	7.87 ± 1.91^ab^	0.121	0.002**	0.2
*IL-4*	*1.89* ± *0.233*^*b*^	*5.33* ± *2.88*^*b*^	*0.687* ± *0.136*^*a*^	*1.99* ± *0.255*^*b*^	< 0.001**	< 0.001**	*0.069*
*IL-10*	*61* ± *55.5*^*b*^	*7.1* ± *1*^*b*^	*2.05* ± *1.06*^*a*^	*5.43* ± *1.31*^*b*^	< 0.001**	< 0.001**	0.045*
*sCD40L*	*2640* ± *641*^*a*^	*885* ± *473*^*b*^	*5390* ± *439*^*a*^	*2370* ± *968*^*b*^	0.03*	< 0.001**	*0.261*
**Macrophage/T-cell recruitment roles**							
MCP-1	368 ± 40.8^ab^	260 ± 37.3^b^	433 ± 31.9^a^	353 ± 46.3^ab^	0.111	0.014*	0.333
*MIP-1α*	*14* ± *8*	*6.31* ± *2.56*	*11.9* ± *1.85*	*21.9* ± *18.1*	*0.182*	*0.066*	*0.659*
IL-13	4 ± 1.78	1.96 ± 0.419	4.49 ± 0.549	2.44 ± 0.555	0.648	0.829	0.707
**Wound healing/miscellaneous**							
TGFα	13.9 ± 8.19^ab^	2.26 ± 0.686^b^	12.2 ± 1.45^a^	5.34 ± 1.68^b^	< 0.001**	< 0.001**	0.893
VEGF	253 ± 112	66.9 ± 32.7	60.5 ± 18.1	181 ± 126	0.078	0.716	0.109
*G-CSF*	*18.1* ± *8.97*	*6.65* ± *4.51*	*4.31* ± *0.586*	*6.25* ± *1.79*	*0.256*	*0.769*	0.044*
IL-5	26.2 ± 20.3^b^	1.2 ± 0^b^	1.38 ± 0.39^a^	1.5 ± 0.462^b^	< 0.001**	0.02*	0.048*

The effect of handling had a greater impact on cytokine expression (significant for nine cytokines), than the impact of sex (significant for six cytokines). The interaction between sex and subject handling was significant for four cytokines. Descriptive statistics are presented as cytokine concentration (pg/mL). Two-way ANOVA was performed using Box-Cox transformed data to meet statistical assumptions. Regular text = accept; italicized = marginal. Cytokines with validation outcome of reject are not included.

Values are means ± SEM.

*p < 0.05; **p < 0.01; ***p < 0.001; ****p < 0.0001.

^a,b^Means that are not significantly different as reported by an all-pairwise comparison are followed by a common superscript letter. Groups were analyzed by two-way ANOVA and the Tukey test.

^1^S1 × S2 = Sex × Handling interaction effect.

## Discussion

Of the 23 cytokines that underwent validation in healthy NHP serum, 11 met all acceptance criteria, 5 were marginally accepted, and 7 were rejected. These results demonstrate that the Millipore Milliplex multiplex assay can be reliably used to detect each of the 11 accepted cytokines (TGFα, IFNγ, IL-2, IL-15, IL-13, IL-5, IL-6, IL-8, MCP-1, IL-12/23 (p40) and VEGF) at endogenous levels in serum from healthy NHPs. The marginal cytokines meeting most of the validation criteria (G-CSF, IL-10, sCD40L, IL-4 and MIP-1α) require consideration of method specific limitations prior to interpretation of absolute measurements. The 7 cytokines in the panel that did not meet our pre-established acceptance criteria (GM-CSF, IL-17A, IL-1ra, IL-1β, TNFα, MIP-1β and IL-18) may not be reliably detected using this method in healthy NHP serum. This validation was performed entirely on frozen samples as is typical with batch assays; further testing to evaluate validation in fresh samples or samples undergoing freeze–thaw cycles are needed. Rejected cytokines were excluded from future analyses in this study since results were deemed invalid.

This study confirms this multiplex assay is capable of detecting specific cytokine concentrations in healthy male and female cynomolgus and rhesus macaques both consciously cooperating and sedated for other procedures and calls attention to the importance of cohort and model-handling experimental parameters when evaluating cytokine levels. We demonstrate significant differences in male vs. female and cooperating vs. sedated cynomolgus macaques, as well as baseline differences between cynomolgus and rhesus macaques. Interestingly, we saw an overall reduction in, or blunting of, cytokine levels after sedation of both male and female cynomolgus macaques. We confirmed that this was due to a physiologic decrease in cytokine levels as opposed to direct assay interference with ketamine by spiking ketamine in vitro into the samples, showing no difference in cytokine levels between unspiked and spiked samples. Of note, cytokine levels were measured immediately upon/during sedation, and evaluation of the short- and long-term effects of sedation will be critical in understanding implications.

The use of a retrospective study design allowed us to aggregate a large NHP data set that is an advantage over the small experimental groups generally used in NHP modeling, however, this approach introduces inherent limitations. We observed wide inter-animal variation in cytokine expression, and while day-to-day within-subject (intra-)animal variability is relatively stable compared to population variability, subsequent follow-up studies could address this in combination with other factors prospectively. Even considering this variability, sedation was still significantly associated with cytokine blunting, suggesting subsequent studies are worthwhile to study the specific impact of each unique regimen on cytokine levels and is intentionally balanced for demographic factors which could not be done retrospectively with this cohort.

This blunted cytokine response after sedation has profound implications in preclinical and clinical research and treatments. A blunted cytokine response could negatively impact clinical outcomes such as wound healing and the ability to overcome infection. However, it also has the possibility for positive impacts on clinical outcomes such as mitigating transplant rejection and reducing the cytokine storm that contributes to tissue damage and end-organ dysfunction in sepsis^32,33^. The blunted cytokine response we demonstrated does not seem to be pathway-specific, as both IL-12/23 (p40) (a potent Th1 polarizing cytokine) and IL-10 (a potent Th2/regulatory cytokine) are reduced after sedation, which highlights the importance of understanding sedation effects in various disease states given the dual effects of Th1- or Th2-driven pathways. To our knowledge, the effects of sedation on cytokine levels in NHPs has not been investigated or described previously. Anesthetics and sedatives such as propofol, dexmedetomidine, and ketamine, which are commonly used in the intensive care setting for the purposes of sedation and pain control for intubated and/or critically ill patients, have also been shown to reduce peripheral cytokine production in humans and rodents, consistent with our findings. Specifically, Propofol and ketamine reduce the production of pro-inflammatory cytokines TNF-a, IL-6, and IL-8 in LPS-challenged in vitro cells as well as in induced rodent models of sepsis^33,34^. Additionally, use of sedatives in rodent models of sepsis has shown improved outcomes such as end-organ dysfunction and mortality, which has been postulated to be due to a reduction in the overwhelming systemic inflammation of sepsis^32,33^. These drug combinations are commonly used in clinical settings both in intensive care and the perioperative period.

We additionally observed overall higher levels of both pro-inflammatory and anti-inflammatory cytokines in female cynomolgus macaques in comparison with male cohorts. These sex differences are especially critical to recognize as preclinical and even clinical studies are often male-skewed, which can dramatically impact efficacy of treatments for female patients. Studies investigating sex differences in the immune response have been sparse, though it is generally accepted that adult female mammals have greater inflammation at baseline than males consistent with our data^35^. Sex-dependent differences in immune responses need to be tested in a more dedicated manner in the future in order to develop therapeutics that are effective for individuals of any sex. A recent study noted that within a sex-balanced study of healthy human serum, there was significant variation between expression levels in males and females, though this variation was not explained by the estrous cycle of female subjects^36^. This emphasizes the importance of designing studies with both baseline controls within subjects, as well as sex-balancing between subjects within a cohort.

In addition to the differences seen between cohorts of sex and sedation status, our data shows a high level of variability between individual animals as demonstrated by large standard deviations despite relatively high sample numbers. Such variability, despite being exposed to similar environmental conditions, may be attributed in part to the outbred nature of primates. Similarly high levels of cytokine variability have also been recognized in other species, including dogs and humans^37,38^. This highlights the difficulty of determining a ‘reference range’ for each cytokine; instead, we advocate for using each animal’s healthy state as a baseline and assessing change over time in response to illness or treatment, a strategy proposed for disease monitoring in human patients. Our stability studies show an average threefold change in all cytokines for samples taken from the same healthy animal on different days. Similar trends and challenges are also seen in human studies. Inconsistencies in cytokine measurements have also been reported in human multiplex kits^17,18^, reporting variable recoveries, indicating that the challenges of measuring cytokines simultaneously in a single matrix are not specific to NHPs. Our findings are similar to those from studies of the stability of endogenous cytokine expression in humans over time, which found a greater variability in cytokine expression between individuals at the same time point, than there was from the same individual across time points. In fact, NHPs are an ideal preclinical model as they mimic the heterogeneity, both inter- and intra-animal, that is seen in human cohorts.

Interpretation of cytokine levels, either individually or in a multiplex assay, has many inherent challenges. Cytokines are, by nature, pleiotropic in their effects^39^, and exhibit dynamic expression patterns that are independent of illness. Expression of various matrix proteins (including blood clotting factors and hormones) have been shown to exhibit diurnal and seasonal fluctuations in humans^40,41^. Such fluctuations may account for the intra- and inter-animal variability observed in this study. Diurnal fluctuations of cortisol have been shown to have an inverse effect on the expression of several inflammatory cytokines (IFNγ, TNFα, IL-1 and IL-12). Humans have lower levels of cortisol expression between the hours of midnight and 5:00 a.m., which may contribute to noted increases in inflammatory symptoms at night^42^. This demonstrated relationship between cortisol and inflammatory cytokines suggests that an increase in cortisol as a response to stress may directly influence the expression of inflammatory cytokines, making this relationship critical to the interpretation of inflammatory markers in animal subjects undergoing handling related stresses.

While we present absolute cytokine levels, it should be mentioned that interpretation requires context because “normal” values have not been well established for most cytokines. As cytokine levels vary by the individual and type of disease being studied, more commonly the balance between cytokines within the overall physiologic system or a change in levels from an individual’s baseline are associated with worsening or improving disease^43^. For example, in one study of neonatal sepsis, cutoffs for IL-6, TNFα, and IL-1β were found to facilitate diagnosis on day one of infection, and combining these multiple cytokines enhanced accuracy in diagnosis, suggesting that all 3 cytokine levels in relation to each other are important in disease pathogenesis. However, these cutoff levels even in well-studied diseases such as sepsis have not yet been implemented routinely in the clinical setting, as additional work is needed to support their utility across populations and also to harmonize test methods^44^. Further, cytokine expression has been shown in humans to be influenced by a variety of other continuously changing demographic characteristics, including both downward (e.g. IL-2Rα) and upward (e.g. IL-17) changes in expression with age^45^. The cohorts used in this study displayed minimal age-related differences in cytokine expression, though this finding is likely explained by an age distribution skewing heavily towards the 4–6 years age range with only a small number of older animals. This range reflects the modeling of young healthy adults, but evidently ongoing studies would benefit from including a cohort of older, adult animals to better explore the effects of age on cytokine expression in macaques especially in studies where animals across the age spectrum are of interest (e.g. obesity, aging, chronic disease).

Multiplexing technology has significant animal welfare and research advantages when compared to traditional methods like ELISA; the ability to simultaneously measure multiple cytokines from a single small volume sample allows for reduced animal handling, reduced blood draw volume, and increased research efficiency. While multiplex assays cost more than individual ELISAs, they are relatively cost-effective when four or more analytes can be accurately measured^46^. Furthermore, the need to evaluate multiple cytokine levels together to better understand the overall clinical picture of a subject makes multiplexing an attractive method. Comparisons of multiplex-based immunoassays with single analyte ELISAs showed that while measurements of high abundance cytokines were highly correlated between methodologies, low abundance cytokines in healthy human subjects (IL-6, TNFα, IL-1β) were poorly correlated^47^. This trade-off between multiplexing and assay sensitivity for low abundance cytokines must be carefully considered when designing a study, and caution should be used when interpreting results near the LLOD of a multiplex assay. The popularity of multiplex assays and the importance of NHPs in pre-clinical testing has resulted in an increase in the number of NHP multiplex cytokine assays on the market. Given the importance of understanding the cytokine response in pre-clinical testing, it will be critical to continue validation efforts of these commonly used assays.

The sample matrix (serum or plasma) can affect the detection of circulating cytokines, specifically those that are expressed near the limit of detection. Non-specific background is significantly increased in serum as compared to plasma^48^, making changes in expression of low-abundance cytokines harder to detect in serum. Differences in the composition of the sample matrix, including the protein-rich environment of biological fluids, may lead to cross-reactivity within the assay, resulting in poor analyte recovery such as was noted in this study (ie. IL-17A, MIP-1β, IL-18). For example, a common plasma protein, alpha-2 macroglobulin, has been shown to interfere in ELISA assays and lower the detection of IL-2, IL-6, IL-4, IL-1β and TNFα by up to 26%^49^. Additionally, autoantibodies can inhibit their cytokines in vitro, leading to artificially low measurements of the targeted analytes^50^. Conversely, artificially high cytokine measurements can result from soluble cytokine receptors being present in the blood matrix^51^. For these reasons, it is critical to consider sample matrix when designing multiplex experiments.

This work demonstrates uniqueness in individual cytokine profiles and the influence of sex, species, and sedation. The NHP provides an ideal model in which to study these questions as both consciously cooperating and sedated animals of both sexes can be sampled, and healthy animals can be compared to a multitude of disease processes of interest. This provides a stepping stone for future work in evaluating population- or subject-based reference intervals for cynomolgus and rhesus macaques. Specifically, understanding the role and mechanisms by which anesthetics and sedatives reduce inflammation will help improve patient outcomes, especially in infectious processes such as sepsis and ARDS from COVID-19.

## Conclusions

Multiplexing cytokine measurements is an important research technique that improves efficiency, preserves biological samples allowing for reduction in number of animal subjects needed, and gives a wealth of complex information about the clinical state of a subject. This study outlines the limitations and challenges involved in detection and interpretation of multiple cytokines in healthy NHP serum, as well as the variability in cytokine expression due to species, sex, and sedation. Of the 23 cytokines validated in healthy NHP serum for the Millipore Milliplex assay, 11 met all acceptance criteria (TGFα, IFNγ, IL-2, IL-15, IL-13, IL-5, IL-6, IL-8, MCP-1, IL-12/23 (p40) and VEGF). Five were marginally accepted as they didn’t meet acceptance criteria for either recovery or linearity (G-CSF, IL-10, sCD40L, IL-4 and MIP-1α). Seven were rejected as they didn’t meet acceptance criteria for both recovery and linearity (GM-CSF, IL-17A, IL-1ra, IL-1β, TNFα, MIP-1β and IL-18), indicating that these analytes may not be reliably detected using this method in healthy NHP serum.

Prior to interpretation of raw multiplex cytokine data, it is critical to identify potential influences on cytokine expression, including species, sex, sedation, stress, and collection method. Following sampling, method-specific limitations to consider include matrix effects, species specific binding, and potential interactions between analytes that are included in a multiplex assay. Validation studies should be undertaken to account for these influences, and subject cohorts should be thoughtfully designed in a sex-balanced manner and with attention to model handling aspects such as sedation to ensure accurate interpretation and reporting of results from multiplex cytokine assays. In our study, we demonstrate the detectable levels and mean levels for cohorts of cynomolgus and rhesus macaques. We also demonstrate overall higher levels in female vs. male cynomolgus macaques and cooperating/awake vs. sedated cynomolgus macaques, which has possible significant clinical implications such as in transplantation and infectious disease modeling.

## Supplementary Information

Supplementary Information.
