# Supplementary material for: Serum cytokine profiles in healthy nonhuman primates are blunted by sedation and demonstrate sexual dimorphism as detected by a validated multiplex immunoassay
Source: Sci Rep. 2021 Jan 27;11:2340. doi: 10.1038/s41598-021-81953-7 (PMC7840937; doi:10.1038/s41598-021-81953-7)
Supplement: Supplementary file 1 — Supplementary Information. [file 41598_2021_81953_MOESM1_ESM.pdf]

Supplement for manuscript:

Serum cytokine profiles in healthy nonhuman primates are blunted by sedation and demonstrate sexual dimorphism as detected by a validated multiplex immunoassay

Authors: Laura Hocum Stone[1,5], Scott Hunter Oppler[1,5], Julia L Nugent[1,5], Sarah Gresch[2,3], Bernhard J. Hering[1], Michael P. Murtaugh[4#], Rebecca L Hegstad-Davies[2], Sabarinathan Ramachandran[1], Melanie L. Graham[1,2*]

Affiliations:

[1] Department of Surgery, University of Minnesota, Minneapolis, MN 55108, USA

[2] Department of Veterinary Population Medicine, University of Minnesota, St. Paul MN 55108, USA

[3] Veterinary Diagnostic Lab, College of Veterinary Medicine, University of Minnesota, St. Paul MN, 55108, USA

[4] Department of Veterinary and Biomedical Sciences, University of Minnesota, St. Paul, MN 55108, USA

[5] These authors contributed equally.

[#] deceased

* Correspondence to: Melanie L. Graham graha066@umn.edu

Supplemental Table 1: Demographics of NHPs.

	Cynomolgus macaques	Rhesus macaques
**Total**	93	26
**Sex**		
Female (n (%))	29 (31)	5 (19)
Male (n (%))	64 (69)	21 (81)
**Age in years** (median (IQR))	5.1 (4.5 - 5.8)	3.9 (3.5 - 4.4)
Range	3.0 - 12.9	1.7 - 13.1
**Weight in kg** (median (IQR))	4.8 (4.1 - 5.7)	6.1 (3.9 - 7.3)
Range	3.1 - 7.9	3.1 - 16.2
**Sedation status**		
Cooperating (n (%))	64 (69)	26 (100)
Sedated (n (%))	29 (31)	0 (0)
Sedated with ketamine (n (%))	27 (93)	n/a
Sedated with ketamine alone (n (%))	5 (18)	
Sedated with ketamine + isoflurane (n (%))	11 (41)	
Sedated with ketamine + isoflurane + midazolam (n (%))	11 (41)	
Sedated with tiletamine + zolazepam + isoflurane (n (%))[a]	2 (7)	

[a]Animals sedated with tiletamine + zolazepam excluded from sedated animal cohort.

Supplemental Table 2: Sample set up for recovery and linearity validation studies in healthy NHP serum.

Sample Source	Sample Type	Final Sample Composition for Recovery Studies	Dilution for Linearity Studies	Assay Plate Setup for Recovery and Linearity Studies

Animal #1	Neat	100% NHP serum	n/a	Each sample tested in triplicate on a single assay plate<br><br>(note that 4 assay plates were needed in total to complete validation)
	Serum + midrange standard	75% NHP serum + 25% midrange standard	n/a	
	Serum + high standard	75% NHP serum + 25% high standard	1:2 and 1:5	
Animal #2	Neat	100% NHP serum	n/a	
	Serum + midrange standard	75% NHP serum + 25% midrange standard	n/a	
	Serum + high standard	75% NHP serum + 25% high standard	1:2 and 1:5	
Animal #3	Neat	100% NHP serum	1:2	
	Serum + midrange standard	75% NHP serum + 25% midrange standard	n/a	
	Serum + high standard	75% NHP serum + 25% high standard	1:2 and 1:5	
Animal #4	Neat	100% NHP serum	1:2	
	Serum + midrange standard	75% NHP serum + 25% midrange standard	n/a	
	Serum + high standard	75% NHP serum + 25% high standard	1:2 and 1:5	
Animal #5 and #6	Neat	100% NHP serum	1:2	
	Serum + midrange standard	75% NHP serum + 25% midrange standard	n/a	
	Serum + high standard	75% NHP serum + 25% high standard	1:2 and 1:5	
Animal #7	Neat	100% NHP serum	n/a	
	Serum + midrange standard	75% NHP serum + 25% midrange standard	n/a	
	Serum + high standard	75% NHP serum + 25% high standard	1:2 and 1:5	
Animal #8	Neat	100% NHP serum	n/a	
	Serum + midrange standard	75% NHP serum + 25% midrange standard	n/a	
	Serum + high standard	75% NHP serum + 25% high standard	1:2 and 1:5	
Animal #9	Neat	100% NHP serum	n/a	
	Serum + midrange standard	75% NHP serum + 25% midrange standard	n/a	
	Serum + high standard	75% NHP serum + 25% high standard	1:2 and 1:5	

Supplemental Table 3. Sample set up for intra-assay (A) and inter-assay (B) precision validation studies in healthy NHP serum.

A.

Sample Source	Sample Type	Final Sample Composition for Intra-assay Precision Studies	Assay Plate Setup for Intra-assay Precision Studies
Pooled serum #2	Serum + midrange standard	>90% NHP serum + <10% midrange standard	Each sample tested in 12 replicates on a single assay plate
	Serum + high standard	>90% NHP serum + <10% high standard	
Pooled serum #3	Serum + midrange standard	>90% NHP serum + <10% midrange standard	
	Serum + high standard	>90% NHP serum + <10% high standard	

B.

Sample Source	Final Sample Composition for Inter-assay Precision Studies	Assay Plate Setup for Inter-assay Precision Studies
Manufacturer control #1	100% control #1	Each sample tested in triplicate on each of the 4 assay plates
Manufacturer control #2	100% control #2	
Pooled serum #2 + manufacturer control #1	50% NHP serum + 50% 1:4 diluted control #1	
Pooled serum #3 + manufacturer control #2	50% NHP serum + 50% 1:4 diluted control #2	

Supplemental Table 4. Sample set up for sensitivity validation studies in healthy NHP serum.

Sample Source	Dilution for Sensitivity Validation Studies	Setup for Sensitivity Validation Studies
Lowest standard	1:2	Each sample tested in 6 replicates on a single assay plate
	1:4	

Supplemental Table 5. Results of quantitative recovery studies. Seven individual animal samples and one NHP serum pool were spiked with known amounts of standard at various levels to yield 17 separate spiked samples, each measured in duplicate, over 3 assays. Each high and low accuracy shown here is the average of duplicates from one of the 17 spiked samples. In order to meet assay acceptance criteria, accuracy is expected to be 75% to 125% of expected values.

Cytokine	Lowest Recovery	Highest Recovery	Median Recovery	Mean Recovery	Millipore Result	Outcome
G-CSF	38%	95%	74%	71%	93%	Fail
GM-CSF	74%	585%	294%	307%	99%	Fail
IFNγ	36%	120%	77%	75%	92%	Pass
IL-10	23%	113%	57%	67%	101%	Fail

IL-12/23(p40)	36%	125%	84%	82%	95%	Pass
IL-13	32%	131%	78%	79%	90%	Pass
IL-15	53%	113%	82%	83%	93%	Pass
IL-17A	8%	78%	57%	47%	93%	Fail
IL-18	2%	31%	23%	20%	91%	Fail
IL-1ra	27%	130%	50%	58%	90%	Fail
IL-1β	27%	115%	61%	63%	91%	Fail
IL-2	55%	139%	91%	93%	90%	Pass
IL-4	0%	67%	45%	45%	94%	Fail
IL-5	57%	136%	109%	106%	96%	Pass
IL-6	48%	237%	125%	118%	87%	Pass
IL-8	50%	143%	96%	91%	89%	Pass
MCP-1	63%	252%	102%	106%	90%	Pass
MIP-1α	14%	152%	63%	65%	89%	Fail
MIP-1β	2%	101%	53%	44%	90%	Fail
sCD40L	43%	108%	87%	86%	90%	Pass
TGFα	8%	204%	81%	82%	98%	Pass
TNFα	17%	139%	69%	96%	96%	Fail
VEGF	55%	110%	88%	86%	70%	Pass

Supplemental Table 6: Summary of quantitative linearity studies of neat samples and samples spiked with the highest assay standard.

Cytokine	Linearity of neat samples *(n=3)*	Linearity of spiked samples *(n=16)*
G-CSF	62%	172%
GM-CSF	< range	166%
IFNγ	112%	128%
IL-10	100%	151%
IL-12/23(p40)	107%	151%
IL-13	98%	95%
IL-15	82%	120%
IL-17A	126%	193%
IL-18	87%	225%
IL-1ra	163%	181%
IL-1β	< range	139%
IL-2	119%	116%
IL-4	248%	111%
IL-5	33%	132%

IL-6	55%	112%
IL-8	125%	110%
MCP-1	105%	99%
MIP-1α	251%	120%
MIP-1β	125%	306%
sCD40L	126%	148%
TGFα	91%	235%
TNFα	71%	162%
VEGF	114%	151%

Supplemental Table 7. Summary of quantitative precision data observed. For intra-assay precision n=24 in one assay. For inter-assay precision n=4 (4 assays in which each control was tested in triplicate). In order to meet assay acceptance criteria, intra-assay precision is expected to be ≤ 20% and inter-assay precision is expected to be ≤ 25%.

Cytokine	Intra-assay Precision (CV %)	Outcome	Inter-assay Precision (CV %)	Outcome
G-CSF	6%	Pass	16%	Pass
GM-CSF	2%	Pass	9%	Pass
IFNγ	2%	Pass	7%	Pass
IL-10	2%	Pass	6%	Pass
IL-12/23(p40)	2%	Pass	11%	Pass
IL-13	1%	Pass	8%	Pass
IL-15	2%	Pass	9%	Pass
IL-17A	1%	Pass	15%	Pass
IL-18	8%	Pass	14%	Pass
IL-1ra	3%	Pass	15%	Pass
IL-1β	2%	Pass	4%	Pass
IL-2	1%	Pass	6%	Pass
IL-4	9%	Pass	7%	Pass
IL-5	3%	Pass	5%	Pass
IL-6	3%	Pass	6%	Pass
IL-8	2%	Pass	7%	Pass
MCP-1	3%	Pass	6%	Pass
MIP-1α	4%	Pass	8%	Pass
MIP-1β	4%	Pass	10%	Pass
sCD40L	7%	Pass	7%	Pass
TGFα	4%	Pass	4%	Pass
TNFα	6%	Pass	45%	Fail
VEGF	4%	Pass	12%	Pass

Supplemental Table 8: Results of sensitivity studies. The lower limit of quantification (LLOQ), lower limit of detection (LLOD), lowest standard and Millipore's reported sensitivity for each analyte are shown. In order to meet assay acceptance criteria, it is expected that the LLOD will be at, or below, the lowest standard for that analyte.

Cytokine	Lowest Standard (pg/mL)	LLOD (pg/mL)	LLOQ (pg/mL)	Vendor Reported Sensitivity (pg/mL)	Outcome
G-CSF	2.4	0.6	2.4	2.1	Pass
GM-CSF	2.4	0.6	2.4	1.8	Pass
IFNγ	2.4	0.6	2.4	1.6	Pass
IL-10	12.2	3.05	12.2	6.4	Pass
IL-12/23(p40)	2.4	2.4	2.4	1.5	Pass
IL-13	2.4	0.6	2.4	5.8	Pass
IL-15	2.4	2.4	2.4	0.5	Pass
IL-17A	2.4	0.6	2.4	1.3	Pass
IL-18	12.2	6.1	12.2	6.1	Pass
IL-1ra	2.4	2.4	2.4	2.4	Pass
IL-1β	2.4	1.2	1.2	1.2	Pass
IL-2	2.4	0.6	2.4	2.1	Pass
IL-4	4.9	4.9	4.9	3.1	Pass
IL-5	2.4	1.2	1.2	1.5	Pass
IL-6	2.4	2.4	2.4	1.6	Pass
IL-8	2.4	1.2	2.4	1.1	Pass
MCP-1	2.4	2.4	2.4	3.1	Pass
MIP-1α	2.4	0.6	2.4	4.9	Pass
MIP-1β	2.4	0.6	0.6	1.6	Pass
sCD40L	2.4	2.4	2.4	2.1	Pass
TGFα	2.4	1.2	1.2	1.1	Pass
TNFα	2.4	0.6	2.4	1.6	Pass
VEGF	2.4	2.4	13.6	13.6	Pass

Supplemental Table 9. Cytokine detection in cooperating and sedated cohorts of cynomolgus macaques. Regular text = accept; italicized = marginal. Cytokines with validation outcome of reject are not included.

Cytokine	Cooperating *(n=64)*	Sedated *(n=27)*	*P* Values
	Median (pg/mL)	Median (pg/mL)	Male vs. Female
**Major Pro-Inflammatory Roles**			
IFNγ	2.9 (1.2 - 7.3)	1.2 (1.2)	0.0049 **

IL-6	1.1 (0.3 - 2.1)	1.2 (1.2)	0.0386<br>*
IL-8	1750.0 (869.8 - 4072.0)	1210.0 (630.4 - 3578.0)	0.3052
IL-12/23 (p40)	77.2 (46.7 - 137.4)	9.0 (1.2 - 69.8)	< 0.0001<br>***
IL-15	2.9 (1.3 - 5.7)	1.2 (1.2 - 3.4)	0.0067<br>**
**Major Anti-Inflammatory Roles**			
IL-2	8.8 (4.9 - 12.3)	4.5 (1.2 - 10.4)	0.0066<br>**
IL-4	*0.3 (0.3 - 2.3)*	*2.5 (2.5)*	*<0.0001<br>****
IL-10	*0.3 (0.3 - 3.6)*	*6.1 (6.1)*	*<0.0001<br>****
sCD40L	*4576.0 (1982.0 - 6413.0)*	*225.3 (77.1 - 1094.0)*	*<0.0001<br>****
**Macrophage/T-Cell Recruitment**			
MCP-1	388.9 (275.4 - 496.9)	295.5 (212.9 - 344.7)	0.0116<br>*
MIP-1α	*8.0 (3.1 - 14.4)*	*1.2 (1.2 - 8.9)*	*0.0155<br>**
IL-13	2.6 (1.5 - 6.1)	1.2 (1.2 - 12.1)	0.0771
**Wound Healing/Miscellaneous**			
TGFα	7.9 (4.3 - 14.3)	1.2 (1.2 - 3.9)	<0.0001<br>***
VEGF	11.6 (4.6 - 110.8)	13.8 (2.3 - 85.4)	0.9604
G-CSF	*3.8 (1.1 - 8.7)*	*1.2 (1.2 - 5.7)*	*0.7904*
IL-5	0.3 (0.3 - 1.2)	1.2 (1.2)	0.0046<br>**

Supplemental Table 10. Cytokine detection in cynomolgus macaque serum neat or ketamine-spiked (2000 ng/mL). Regular text = accept; italicized = marginal. Cytokines with validation outcome of reject are not included.

Cytokine	Neat *(n=3)*		Ketamine-Spiked *(n=3)*		*P*-Values
	Range (pg/mL)	Mean ± SD (pg/mL)	Range (pg/mL)	Mean ± SD (pg/mL)	Neat v. Spiked
**Major Pro-Inflammatory Roles**					
IFNγ	1.2 - 10.4	4.3 ± 5.3	1.2 - 7.1	3.2 ± 3.4	>0.99
IL-6	1.2 - 3.2	1.9 ± 1.2	1.2 - 2.5	1.6 ± 0.7	>0.99
IL-8	1639 - 7578	4174 ± 3063	1778 - 5871	3368 ± 2194	0.5
IL-12/23 (p40)	6.3 - 59.1	25.9 ± 29.0	1.2 - 44.8	17.3 ± 24.0	0.25
IL-15	1.2 - 3.4	2.5 ± 1.2	1.2 - 2.8	2.2 ± 0.9	0.5
**Major Anti-Inflammatory Roles**					
IL-2	3.6 - 10.5	6.0 ± 3.9	4.0 - 10.5	6.2 ± 3.8	0.5
IL-4	*2.5*	*2.5 ± 0*	*2.5*	*2.5 ± 0*	*>0.99*
IL-10	*4.7 - 6.1*	*5.6 ± 0.8*	*2.4 - 6.1*	*4.1 ± 1.9*	*0.5*
sCD40L	*860.5 - 945.4*	*907.3 ± 43.1*	*658.8 - 1078*	*850.3 ± 212.0*	*0.75*

**Macrophage/T-Cell Recruitment Roles**					
MCP-1	273.5 - 465.4	364.3 ± 96.4	235.0 - 458.1	371.6 ± 119.7	>0.99
MIP-1α	*1.2 - 13.3*	*6.2 ± 6.3*	*1.2 - 10.7*	*4.9 ± 5.1*	*0.5*
IL-13	1.2 - 25.0	12.7 ± 11.9	1.2 - 25.3	9.2 ± 13.9	>0.99
**Wound Healing/Miscellaneous**					
TGFα	1.2	1.2 ± 0	1.2	1.2 ± 0	>0.99
VEGF	1.2 - 124.6	42.4 ± 71.3	1.2 - 124.6	42.4 ± 71.3	>0.99
G-CSF	*8.9 - 130.0*	*69.5 ± 85.6*	*5.5 - 105.9*	*40.7 ± 56.5*	*0.8*
IL-5	1.2	1.2 ± 0	1.2	1.2 ± 0	>0.99
